# Case–control study of the correlation between the five times sit to stand and 6-min walk distance in patients with pancreatic cancer

**DOI:** 10.1007/s00520-022-07402-x

**Published:** 2022-10-28

**Authors:** Yuki Nakashima, Daisuke Iwaki, Toshihiro Kawae, Kenichi Fudeyasu, Kenichiro Uemura, Hiroaki Kimura

**Affiliations:** 1 Division of Rehabilitation, Department of Clinical Practice and Support, Hiroshima University Hospital, Hiroshima, Japan; 2 Department of Physical Therapy, Makuhari Human Care Faculty, Tohto University, Chiba, Japan; 3 Department of Surgery, Graduate School of Biomedical and Health Science, Hiroshima University, Hiroshima, Japan; 4 Department of Rehabilitation, Hiroshima University Hospital, Hiroshima, Japan

**Keywords:** Pancreatic cancer, Surgery, 6-min walk distance, Sit to stand, Hand grip strength

## Abstract

**Purpose:**

Cases of pancreatic cancer are increasing, and the risk of developing this disease reportedly increases with age. In recent years, there has been an increasing number of reports on physical function in patients with pancreatic cancer. Methods such as the 6-min walk distance (6 MWD) should be established to evaluate physical function, as a decline in exercise capacity is an important index in these patients. Recently, the 6 MWD has also been used to evaluate physical function in patients with pancreatic cancer. In healthy older adults, a decrease in 6 MWD is reportedly associated with intrinsic capacity and health status. Such factors make assessing 6 MWD important. However, the measurement of 6 MWD requires a sizable measurement environment. The five times sit to stand (FTSTS) test is a simple method that can be performed using a chair. FTSTS is hypothesized to be a useful assessment scale in patients with pancreatic cancer because it is easy to estimate the decline in physical function in clinical practice if the decline in 6 MWD can be estimated by evaluating FTSTS. The study’s purpose was to clarify this hypothesis and ascertain the cutoff required to determine the decrease in 6 MWD in clinical practice.

**Methods:**

Sixty consecutive patients with preoperative pancreatic cancer who were assessed for physical function were studied. 6 MWD (< 400 m) was the objective variable, and binary logistic regression analysis was performed, with age, BMI, sex, FTSTS, and HGS as explanatory variables. Receiver-operating characteristic (ROC) curve analysis was performed for the explanatory variables, which were found to be significant based on logistic regression analysis. The area under the curve (AUC) was also calculated. Sensitivity, specificity, negative predictive value (NPV), and positive predictive value (PPV) were evaluated. This study was approved by Hiroshima University Hospital’s ethics committee (approval number: E808-1).

**Results:**

Fifty-seven of the 60 patients were included in the analysis. Logistic regression analysis showed that FTSTS was a significant explanatory variable; ROC curve analysis showed an AUC of 0.872 and a cutoff value of 8.98 s. The sensitivity, specificity, PPV, and NPV were 82.4%, 80.0%, 63.6%, and 91.4%, respectively.

**Conclusions:**

A decrease in 6 MWD in preoperative pancreatic cancer patients can be identified by performing FTSTS.

## Introduction

Cases of pancreatic cancer are on the rise, and the risk of developing this disease has been reported to increase with age [1, 2]. Patients with pancreatic cancer often suffer from sarcopenia, with the percentage reported to be as high as 17–65% in previous studies. Sarcopenia is associated with decreased motor function. [3, 4]. In other words, an increasing number of elderly patients with pancreatic cancer are prone to loss of skeletal muscle mass and function.

In recent years, the number of reports on physical function in patients with pancreatic cancer has increased. A decrease in 6-min walk distance (MWD) has been shown to be associated with a decrease in health-related quality of life in patients with pancreatic cancer [5]. Methods such as the 6 MWD must be established to evaluate physical function, as a decline in exercise capacity is an important index in these patients [6]. In recent years, 6 MWD has also been used to evaluate physical function in patients with pancreatic cancer [7–10]. In healthy older adults, a decrease in 6 MWD has been reported to be associated with intrinsic capacity and health status [11, 12]. In preoperative abdominal surgery patients, exercise capacity has been reported to be associated with the occurrence of postoperative complications [13–15]. Such factors make the assessment of 6 MWD important.

However, a 30-m (m) walking path is usually required to measure 6 MWD, and its absence may make measurement difficult because the layout of the walking path may affect performance. The sit-to-stand test has become widely used as a simple method to assess physical function in clinical practice for patients with pancreatic cancer [9, 16]. The five times sit to stand (FTSTS) test is a simple method that can be performed using a chair. In a recent systematic review, FTSTS has been proven to be highly reliable in both healthy adults and those with pathologies [17]. Associations between 6 MWD and orthostatic testing have been reported in healthy adults [18] and in patients with pulmonary disease [19] and cancer [20]. In healthy adults, 6 MWD has been reported to correlate significantly with orthostatic sitting, indicating that the sit-to-stand test (STS) may be a quick surrogate measure of physical and functional capacity. Furthermore, a significant correlation between 6MWD and STS has been shown in patients with pulmonary disease [19] and breast cancer [20].

Therefore, if the association between FTSTS and 6MWD can be clarified in pancreatic cancer patients as it has been clarified in healthy subjects and pulmonary disease, it would be more useful as an evaluation index because it would allow easy estimation of decline in physical function in clinical settings. To the best of our knowledge, no study has investigated the relationship between FTSTS and the decrease in 6 MWD in patients with pancreatic cancer. Therefore, this study aimed to clarify this and to ascertain the FTSTS cutoff required to determine the decrease in 6 MWD in clinical practice.

## Materials and methods

### Study design and sample

All information for this retrospective study was extracted from medical records. Sixty consecutive patients with pancreatic cancer on the waiting list for surgery who were referred to the Department of Rehabilitation Medicine at Hiroshima University Hospital for a physical function evaluation between March 2020 and July 2021 were included in the study. The inclusion criteria for this study were that the patients be diagnosed with pancreatic cancer and scheduled for surgery. Exclusion criteria included patients who needed assistance to walk, patients with severe leg pain or obvious neuropathy that could potentially strongly affect their physical function, and patients with missing motor function values. All assessments of physical function were performed by a physical therapist. Age, body mass index (BMI), sex, and diagnosis were extracted from medical records. This study was approved by the ethics committee of Hiroshima University Hospital (approval number: E808-1; approval date: January 24, 2020).

### Outcome

#### 6 MWD

The patients’ 6 MWD was calculated as the distance walked for 6 min (min). The guidelines of the American Thoracic Society Statement [21] were followed for measurement, and a 30-m walking path was created in an indoor corridor. Cones were placed at the start and at the 30-m point to indicate the turn-around point. The subjects were instructed to walk for as far as possible within 6 min of starting the test. The definition of a 6 MWD decline is now 400 m or less based on previous studies [22, 23].

#### FTSTS

FTSTS repeats the movements of sitting in and standing up from a chair of standard height (40 cm) five times, reproducing the movements as fast as possible [24]. The measurement began at the examiner’s signal, and the time between the initial sitting position and the fifth standing position was measured.

#### Hand grip strength (HGS)

HGS was measured using a Jamar-type digital handgrip dynamometer (MG-4800; CHARDER, Taichung, Taiwan) [25]. The HGS of the left and right hands was measured in a normal sitting position. The elbow was placed flexed at 90°. The maximum value obtained was used in the analysis.

### Sample size calculations and statistical methods

Easy ROC, which is a web tool for receiver-operating characteristic (ROC) curve analysis (version 1.3.1; http://www.biosoft.hacettepe.edu.tr/easyROC/), was used to calculate the sample size. The sample size was estimated to be 54 (estimate area under the curve [AUC] of 0.7, 80% power, and an allocation ratio of 2:1).

All continuous variables are presented as median values (interquartile range). The association between decreased 6 MWD (< 400 m) and FTSTS was examined in this study. Spearman’s rank correlation coefficient was used to investigate the association between 6 MWD and age, FTSTS, HGS, and BMI. Binary logistic regression analysis was performed to adjust for potentially confounding variables such as age, sex, BMI, and HGS to investigate factors contributing to a decrease in the 6 MWD. The cutoff FTSTS value that best predicted the presence or absence of 6 MWD (< 400 m) was determined using the Youden index. The Youden index (maximum [sensitivity + specificity -1]) was used to calculate the cutoff value [26]. Sensitivity, specificity, negative predictive value (NPV), and positive predictive value (PPV) were used to measure diagnostic accuracy. These tools were all used to measure diagnostic accuracy [27]. The ROC curve analysis and AUC were calculated (0.7 ≤ AUC < 0.8 = acceptable discrimination, 0.8 ≤ AUC ≤ 0.9 = excellent discrimination, and 0.9 ≤ AUC = outstanding discrimination). Statistical significance was set at *P* < 0.05.

## Results

**Fig. 1:**
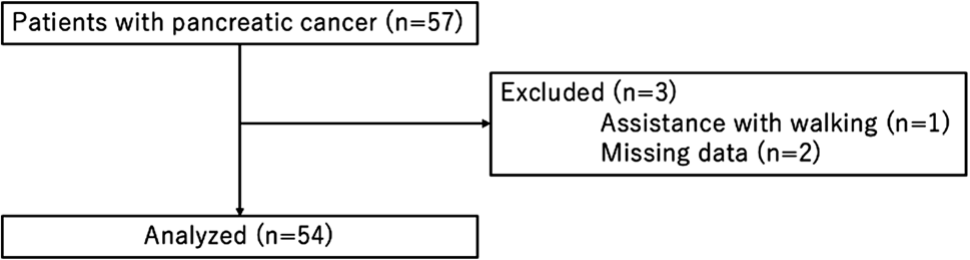
Flowchart of study participants

Fifty-seven patients with pancreatic cancer who met the inclusion criteria were included in the analysis (one patient required assistance with walking, and two patients had missing data; Fig. 1). No adverse events were recorded during the measurement.

The median age was 74 years (interquartile range 66 to 78), and BMI was 21.1 kg/m^2^ (interquartile range 19.2 to 23.2). Forty-eight percent of the patients included were female. The median physical function was 469 m (interquartile range 383 to 518) for 6 MWD, 7.49 s (interquartile range 6.31 to 9.54) for FTSTS, and 22.8 kg (interquartile range 17.5 to 33.1) for HGS (Table 1). Seventeen patients (30%) had decreased 6 MWD (< 400 m).

**Table 1:** Characteristics of the patients

Age (y.o.)	74 (66 to 78)
BMI (kg/m^2^)	21.1 (19.2 to 23.2)
SEX (male/female) (%)	28 (52)/26 (48)
6MWD (m)	469 (383 to 518)
FTSTS (s)	7.49 (6.31 to 9.54)
HGS (kg)	22.8 (17.5 to 33.1)

Median (interquartile range), *BMI*, body mass index; *6 MWD*, 6-min walk distance; *FTSTS*, five times sit to stand; *HGS*, hand grip strength

**Fig. 2:**
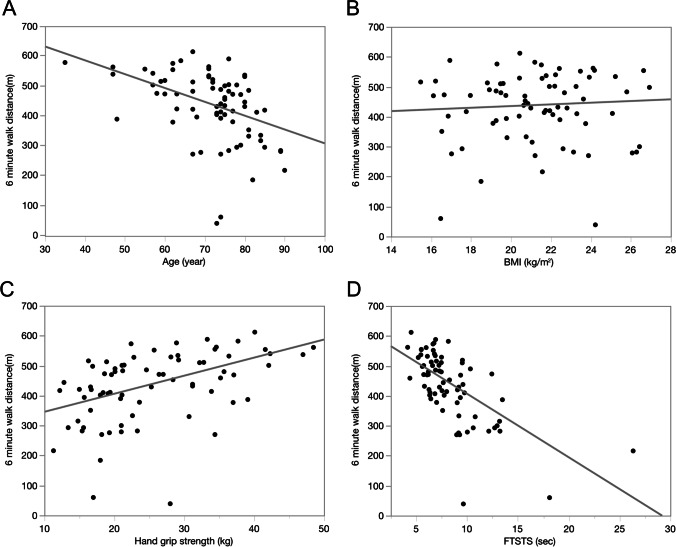
Relationships between 6MWD and age, BMI, HGS, and FTSTS. **A** Relationship between age and 6MWD (*r* =  − 0.47, *P* < .001). **B** Relationship between BMI and 6MWD (*r* = 0.04, *P* = 0.76). **C** Relationship between HGS and 6MWD (*r* = 0.53, *P* < .001). **D** Relationship between FTSTS and 6MWD (*r* =  − 0.63, *P* < .001). 6 MWD, 6-min walk distance; BMI, body mass index; FTSTS, five times sit to stand; HGS, hand grip strength. *P*-values were calculated using Spearman’s rank correlation coefficient

**Fig. 3:**
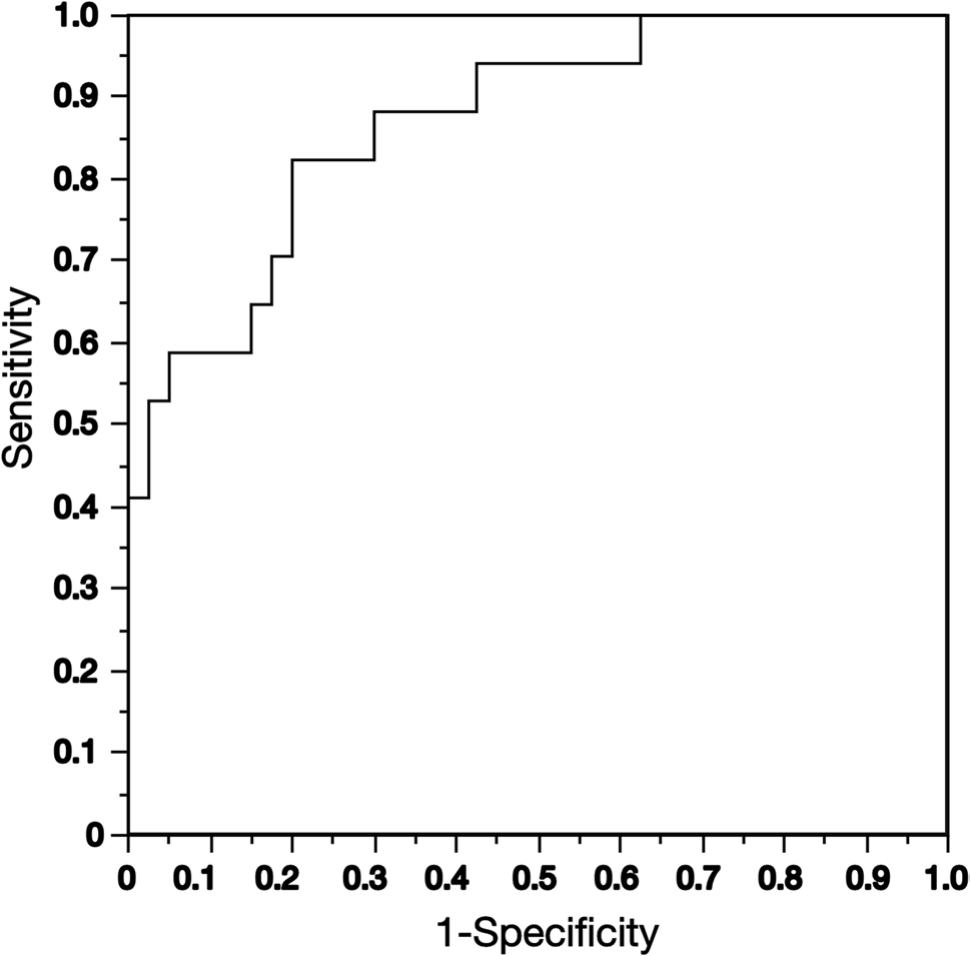
ROC curve analysis of the FTSTS for 6MWD (< 400 m). ROC, receiver-operating characteristic; FTSTS, five times sit to stand test; 6MWD, 6-min walk distance

Age, HGS, and FTSTS indicated significant correlations with 6 MWD (Fig. 2). FTSTS was associated with a decrease in 6 MWD (< 400 m) with an adjusted odds ratio of 2.00 (95% CI: 1.34–2.99, *P* < 0.001; Table 2). Age, sex, BMI, and HGS were not associated with the risk of 6 MWD (< 400 m). ROC analysis showed that the FTSTS time had 82.4% sensitivity, 80.0% specificity, 63.6% PPV, and 91.4% NPV, with an AUC of 0.872 and a cutoff value of 8.98 s (s), which could discriminate the decline in 6 MWD (Fig. 3).

**Table 2:** Results of the binary logistic regression analysis for 6 MWD (< 400 m)

	Odds ratio	95% CI	*P-*value
FTSTS	2.00	1.34 to 2.99	< 0.001*
Age	1.05	0.95 to 1.17	0.36
HGS	0.96	0.83 to 1.12	0.63
BMI	0.96	0.75 to 1.25	0.76
Sex	0.55	0.05 to 5.93	0.62

6 *MWD*, 6-min walk distance; *FTSTS*, five times sit to stand; *HGS*, hand grip strength; *BMI*, body mass index. *P-*values were calculated using a binary logistic regression analysis. *P < 0.05

## Discussion

This study aimed to clarify the association between the decrease in 6 MWD and FTSTS and to determine the cutoff to estimate the decrease in 6 MWD.

The 6 MWD of healthy subjects has been shown to correlate with age [28, 29]. Additionally, a study of hospitalized patients showed that 6 MWD tends to decrease in those with reduced HGS [30]. This study’s results showed that, similar to these previous studies, age, muscle strength, and 6 MWD were significantly associated with 6 MWD and not with BMI [31].

This study also clarified the relationship between 6 MWD (< 400 m) and FTSTS. In this regard, 6 MWD has been reported to be associated with lower limb muscle strength in patients with pancreatic cancer [5], and FTSTS is an indicator of lower limb muscle strength, so the significant association between FTSTS and 6 MWD in this study is also reasonable. Furthermore, HGS is one of the criteria for sarcopenia as well as elevation [32] but may not be sufficient to infer a decline in 6 MWD. Previous studies on 6 MWD and rise time have investigated COPD patients and reported that 6 MWD is associated with FTSTS [19]. Therefore, evaluating FTSTS is important, as it reflects walking ability as well as HGS in patients with pancreatic cancer. In healthy older people, the mean 6 MWD has been reported to be 534–631 m [33], which is higher than this study’s data (median 464 m). This study’s results for the 6 MWD were similar to those of a previous study on patients with pancreatic cancer (mean 463 m) [8]. This study showed that a decrease in 6 MWD (< 400 m) occurred in 30% of patients with pancreatic cancer before surgery, indicating that 6 MWD may already have decreased in patients with pancreatic cancer before surgery. Decreased exercise capacity before pancreatic resection has been reported to be associated with the risk of postoperative complications and a poor prognosis [34–36]. A decrease in 6 MWD (< 400 m) has been reported to increase the risk of postoperative complications [36], and in recent years, prehabilitation to improve physical function and nutritional status, such as exercise and nutritional therapy, has been performed before surgery [16, 37]. A recent systematic review reported that cardiopulmonary exercise testing and 6 MWD are associated with death [38]. Therefore, evaluation of 6 MWD is considered important. However, in clinical practice, the measurement of cardiorespiratory fitness is difficult because of the need for a 30-m walking path and personal limitations. FTSTS has been used as a simple assessment of physical functions as it can be performed in a small space [32]. In an observational study, Karlsson et al. reported that the risk of postoperative complications increased with more time taken from sit to stand [37]. In healthy older subjects, FTSTS averaged 8.63 s and was reported to be lower than that of younger subjects [39]. This study’s FTSTS results were comparable to those of these older adults.

The investigation performed in the study demonstrated that an FTSTS result of 8.98 s was the cutoff to estimate 6 MWD (< 400 m). The results indicate that FTSTS is a simple physical function assessment that can be used in clinical practice to estimate the decline in 6 MWD in patients with pancreatic cancer. A previous study on 6 MWD and STS in women with breast cancer indicated a significant correlation [40]. This could be a useful method of assessment for busy clinicians and physical therapists to understand the decline in patients’ physical function.

The limitations of this study are twofold. First, it was a single-center case–control study. Therefore, a possibility of bias remained in the target population. A multicenter study is required in the future. However, because pancreatic cancer is deadly [41], these data are valuable. Second, this study did not investigate FTSTS in terms of postoperative complications and prognosis. Future prospective research studies that include the impact of FTSTS results on important clinically outcomes are needed. However, an FTSTS result of longer duration is associated with a shorter 6 MWD and may be useful in clinical practice as a simple method to assess physical function in patients with pancreatic cancer.

In conclusion, this study analyzed the factors associated with 6 MWD (< 400 m) in pancreatic cancer patients who were on the waiting list for surgery. Binary logistic regression analysis revealed that rise time was associated with a decrease in 6 MWD. The results of ROC curve analysis also showed that the measurement of FTSTS was an excellent indicator to estimate the decline in 6 MWD. Assessment of the ability of patients with pancreatic cancer via the sit-to-stand test is a simple method of assessment. Additional studies investigating the association between FTSTS and postoperative complications are needed in the future.

## Data Availability

Data is available upon request.

## References

[1] Rawla P, Sunkara T, Gaduputi V (2019). Epidemiology of pancreatic cancer: global trends, etiology and risk factors. World J Oncol.

[2] Pourhoseingholi MA, Ashtari S, Hajizadeh N (2017). Systematic review of pancreatic cancer epidemiology in Asia-Pacific Region: major patterns in GLOBACON 2012. Gastroenterol Hepatol Bed Bench.

[3] Peng Y-C, Wu C-H, Tien Y-W (2021). Preoperative sarcopenia is associated with poor overall survival in pancreatic cancer patients following pancreaticoduodenectomy. Eur Radiol.

[4] Carrara G, Pecorelli N, De Cobelli F (2017). Preoperative sarcopenia determinants in pancreatic cancer patients. Clin Nutr.

[5] Nakashima Y, Kawae T, Iwaki D (2021). Changes in motor function and quality of life after surgery in patients with pancreatic cancer. Eur J Cancer Care.

[6] Agarwala P, Salzman SH (2020). Six-minute walk test: clinical role, technique, coding, and reimbursement. Chest.

[7] Naito T, Mitsunaga S, Miura S (2019). Feasibility of early multimodal interventions for elderly patients with advanced pancreatic and non-small-cell lung cancer. J Cachexia Sarcopenia Muscle.

[8] Solheim TS, Laird BJA, Balstad TR (2017). A randomized phase II feasibility trial of a multimodal intervention for the management of cachexia in lung and pancreatic cancer. J Cachexia Sarcopenia Muscle.

[9] Ngo-Huang A, Parker NH, Bruera E (2019). Home-based exercise prehabilitation during preoperative treatment for pancreatic cancer is associated with improvement in physical function and quality of life. Integr Cancer Ther.

[10] Clauss D, Tjaden C, Hackert T (2017). Cardiorespiratory fitness and muscle strength in pancreatic cancer patients. Support Care Cancer.

[11] Tay L, Tay EL, Mah SM et al (2022) Association of intrinsic capacity with frailty, physical fitness and adverse health outcomes in community-dwelling older adults. J Frailty Aging: 1–9. 10.14283/jfa.2022.28 10.14283/jfa.2022.28 PMC8966852 36629078

[12] Bautmans I, Lambert M, Mets T (2004). The six-minute walk test in community dwelling elderly: influence of health status. BMC Geriatr.

[13] Ramos RJ, Ladha KS, Cuthbertson BH (2021). Association of six-minute walk test distance with postoperative complications in non-cardiac surgery: a secondary analysis of a multicentre prospective cohort study. Can J Anaesth.

[14] Awdeh H, Kassak K, Sfeir P (2015). The SF-36 and 6-minute walk test are significant predictors of complications after major surgery. World J Surg.

[15] Hayashi K, Fukumoto K, Yokoi K (2018). Post-operative delayed ambulation after thymectomy is associated with pre-operative six-minute walk distance. Disabil Rehabil.

[16] Ngo-Huang A, Parker NH, Wang X (2017). Home-based exercise during preoperative therapy for pancreatic cancer. Langenbeck’s Arch Surg.

[17] Muñoz-Bermejo L, Adsuar JC, Mendoza-Muñoz M et al (2021) Test-retest reliability of five times sit to stand test (FTSST) in adults: a systematic review and meta-analysis. Biology (Basel) 10(6):510. https://www.mdpi.com/2079-7737/10/6/510 10.3390/biology10060510 PMC8228261 34207604

[18] Gurses HN, Zeren M, Denizoglu Kulli H, Durgut E (2018). The relationship of sit-to-stand tests with 6-minute walk test in healthy young adults. Medicine (Baltimore).

[19] Meriem M, Cherif J, Toujani S, Ouahchi Y, Hmida AB, Beji M (2015). Oct-Dec) Sit-to-stand test and 6-min walking test correlation in patients with chronic obstructive pulmonary disease. Ann Thorac Med.

[20] Díaz-Balboa E, González-Salvado V, Rodríguez-Romero B (2022). Thirty-second sit-to-stand test as an alternative for estimating peak oxygen uptake and 6-min walking distance in women with breast cancer: a cross-sectional study. Support Care Cancer.

[21] American Thoracic Society Board of Directors (2013). ATS statement. Am J Respir Crit Care Med.

[22] Win T, Jackson A, Groves AM (2006). Comparison of shuttle walk with measured peak oxygen consumption in patients with operable lung cancer. Thorax.

[23] Przybyłowski T, Tomalak W, Siergiejko Z (2015). Polish Respiratory Society guidelines for the methodology and interpretation of the 6 minute walk test (6MWT). Adv Respir Med.

[24] Guinan EM, Doyle SL, Bennett AE (2018). Sarcopenia during neoadjuvant therapy for oesophageal cancer: characterising the impact on muscle strength and physical performance. Support Care Cancer.

[25] Santagnello SB, Martins FM, De Oliveira N, Junior G (2020). Improvements in muscle strength, power, and size and self-reported fatigue as mediators of the effect of resistance exercise on physical performance breast cancer survivor women: a randomized controlled trial. Support Care Cancer.

[26] Takahashi M, Maeda K, Wakabayashi H (2018). Prevalence of sarcopenia and association with oral health-related quality of life and oral health status in older dental clinic outpatients. Geriatr Gerontol Int.

[27] Youden WJ (1950). Index for rating diagnostic tests. Cancer.

[28] Šimundić AM (2009). Measures of diagnostic accuracy: basic definitions. EJIFCC.

[29] Troosters T, Gosselink R, Decramer M (1999). Six minute walking distance in healthy elderly subjects. Eur Respir J.

[30] Shrestha SK, Srivastava B (2015). Six minute walk distance and reference equations in normal healthy subjects of Nepal. Kathmandu Univ Med J (KUMJ).

[31] Martín-Ponce E, Hernández-Betancor I, González-Reimers E (2014). Prognostic value of physical function tests: hand grip strength and six-minute walking test in elderly hospitalized patients. Sci Rep.

[32] Fernandes L, Mesquita AM, Vadala R, Dias A (2016). Reference equation for six minute walk test in healthy Western India population. J Clin Diagn Res.

[33] Chen L-K, Woo J, Assantachai P (2020). Asian Working Group for Sarcopenia: 2019 consensus update on sarcopenia diagnosis and treatment. J Am Med Dir Assoc.

[34] e-stat Retrieved from https://www.e-stat.go.jp/dbview?sid=0003288734. Accessed 29 Jan 2022

[35] Ausania F, Snowden CP, Prentis JM (2012). Effects of low cardiopulmonary reserve on pancreatic leak following pancreaticoduodenectomy. Br J Surg.

[36] Chandrabalan VV, McMillan DC, Carter R (2013). Pre-operative cardiopulmonary exercise testing predicts adverse post-operative events and non-progression to adjuvant therapy after major pancreatic surgery. HPB.

[37] Hayashi K, Yokoyama Y, Nakajima H (2017). Preoperative 6-minute walk distance accurately predicts postoperative complications after operations for hepato-pancreato-biliary cancer. Surgery.

[38] Karlsson E, Egenvall M, Farahnak P (2018). Better preoperative physical performance reduces the odds of complication severity and discharge to care facility after abdominal cancer resection in people over the age of 70 - A prospective cohort study. Eur J Surg Oncol.

[39] Ezzatvar Y, Ramírez-Vélez R, Sáez de Asteasu ML (2021). Cardiorespiratory fitness and all-cause mortality in adults diagnosed with cancer systematic review and meta-analysis. Scand J Med Sci Sports.

[40] Klukowska AM, Staartjes VE, Vandertop WP, Schröder ML (2021). Five-repetition sit-to-stand test performance in healthy individuals: reference values and predictors from 2 prospective cohorts. Neurospine.

[41] Roth MT, Berlin JD (2018). Current concepts in the treatment of resectable pancreatic cancer. Curr Oncol Rep.

